# pH-Dependent Drug Delivery Systems for Ulcerative Colitis Treatment

**DOI:** 10.3390/pharmaceutics17020226

**Published:** 2025-02-10

**Authors:** Yana Gvozdeva, Radiana Staynova

**Affiliations:** 1 Department of Pharmaceutical Sciences, Faculty of Pharmacy, Medical University of Plovdiv, 4002 Plovdiv, Bulgaria; 2 Research Institute, Medical University of Plovdiv, 4002 Plovdiv, Bulgaria; 3 Department of Organisation and Economics of Pharmacy, Faculty of Pharmacy, Medical University of Plovdiv, 4002 Plovdiv, Bulgaria; radiana.staynova@mu-plovdiv.bg

**Keywords:** ulcerative colitis, pH-dependent drug delivery systems, pH-responsive polymers, pH-sensitive carrier, colon-targeted drug delivery

## Abstract

Inflammatory bowel diseases (IBDs), such as ulcerative colitis (UC) or Crohn’s disease, are becoming a growing global problem due to the limitations of current treatments, which fail to address the needs of patients effectively. UC is characterized by the widespread inflammation of the mucosal lining, affecting both the rectum and the entire length of the colon. Over the past forty years, traditional treatments for IBDs have primarily relied on anti-inflammatory drugs and immunosuppressive medications. Treatment could be more effective if drugs could be specifically targeted to act directly on the colon. Conventional drug delivery systems for IBDs encounter numerous challenges on their way to the colon, such as physiological barriers and disease severity. To address these issues, pH-dependent carriers have emerged as a promising advancement, offering a more effective and tolerable treatment for UC. These carriers enable localized, targeted action, reducing side effects and preventing the premature clearance of drugs from inflamed colon tissues. pH-responsive systems are a leading approach for targeted drug release in colitis treatment as they take advantage of the varying pH levels throughout the gastrointestinal tract (GIT). By incorporating pH-sensitive polymers, they ensure drug protection and controlled release in the lower GIT. This review will discuss the advantages and limitations of pH-dependent drug delivery systems for colon-targeted drug delivery.

## 1. Introduction

Targeted drug delivery to the colon is gaining popularity, particularly for treating diseases of the large bowel and enabling the systemic absorption of peptide- and protein-based drugs. Inflammatory bowel diseases (IBDs), including ulcerative colitis (UC) and Crohn’s disease, require localized drug delivery to the colon for effective treatment [1]. Colonic drug delivery can be achieved through oral or rectal administration. The rectal route of drug administration is considered the most direct method for targeting drugs to the colon. However, rectal dosage forms, such as enemas and suppositories, often show limited effectiveness due to significant variability in how the drug is distributed when administered this way [2]. As a result, the oral route is generally preferred. Oral administration is an appealing method for delivering drugs targeted to the colon due to its simplicity, patient comfort, and cost-effectiveness. However, despite being the preferred route, it presents significant challenges. Biopharmaceuticals are prone to degradation in the gastrointestinal tract (GIT) and face difficulty in penetrating the mucus. These issues demand the development of advanced formulation technologies to protect drugs from chemical and enzymatic breakdown in the stomach and small intestine while also improving their ability to permeate mucus for the effective treatment of UC [3].

The colon presents unique benefits as a drug delivery site due to its nearly neutral pH, extended transit time, lower levels of proteolytic enzymes, and higher sensitivity to absorption enhancers. These factors make the distal portion of the GIT particularly favorable for delivering a variety of drug molecules, such as proteins and peptides. To ensure targeted drug release, colon-specific delivery systems must avoid premature release in the upper GIT and rely on a mechanism that triggers a rapid release upon reaching the colon [4].

The main challenge with oral drug delivery to the colon is preventing the drug from being absorbed or degraded in the upper GIT. Overcoming this barrier is crucial for ensuring successful drug delivery to the colon [5]. Why is colon-targeted drug delivery for UC necessary? Because it allows for direct treatment at the site of the disease, reduces the dosage, and minimizes systemic side effects [6].

The primary goal of drug-targeting strategies in IBD is to selectively deliver and concentrate active agents in inflamed intestinal tissues, thereby enhancing therapeutic effectiveness while minimizing side effects. Such targeted delivery systems must be fully biodegradable and highly biocompatible, without any pro-inflammatory effects. Furthermore, these systems should be developed in oral dosage forms to support and maintain patient adherence [7].

Several approaches and strategies have been developed to achieve targeted drug delivery to the colon. These include the design of prodrugs, the use of pH-sensitive polymer coatings, implementing timed-release systems, pressure-controlled or osmotic pressure-controlled drug delivery systems, and others [8]. Coating drugs with pH-sensitive polymers is a simple yet effective method for ensuring colon-specific drug delivery. A key drawback of the prodrug strategy is its limited versatility, mainly due to its reliance on specific functional groups present in the drug moiety for chemical bonding. This dependency limits its applicability to a wide range of drug types. Additionally, prodrugs are considered new chemical entities, which means they must undergo rigorous evaluation before being used as carriers for drug delivery. Their introduction requires careful examination to confirm their safety and effectiveness in transporting medications [9]. Most methods for achieving targeted drug delivery to the colon require specialized equipment, intricate manufacturing processes, and expensive polymers. For example, in the treatment of UC, osmotic-controlled delivery systems are designed with compartments that release the drug at intervals of three to four hours after a gastric delay, preventing early drug release into the small intestine. Once in the colon, the drug is released steadily over a period of 24 h or a shorter duration of 4 hours. On the other hand, time-controlled delivery systems encounter several challenges, such as fluctuations in gastrointestinal movement, including peristalsis and accelerated transit through different sections of the GIT, as well as the gastric emptying time—although minimal—which can affect the efficiency of these systems [10]. There are a number of studies on obtaining pH-dependent drug delivery systems for various diseases, and there are already manufactured drug products that are successfully used in the treatment of UC.

pH-dependent drug delivery systems offer several benefits, including site-specific drug release, the protection of drugs in unfavorable pH conditions, and enhanced therapeutic outcomes. By ensuring precise drug targeting, they minimize side effects and improve efficacy, delivering medication to the intended site at the optimal time. These systems are widely utilized in oral drug delivery, colon-targeted therapy, cancer treatment, and controlled-release formulations [11].

The objective of this review is to examine pH-dependent drug delivery systems, focusing on their advantages and limitations and the application of pH-sensitive polymers, and providing examples from the scientific literature and commercially available products for the treatment of UC.

## 2. GIT Conditions

The GIT is composed of the stomach, small intestine, and large intestine. The large intestine (approximately 1.5 m long in humans), which stretches from the end of the ileum to the anus, is divided into three primary sections: the colon, the rectum, and the anal canal [12].

The colon, which comprises the upper five feet of the large intestine, is primarily located in the abdomen. It is a cylindrical tube lined with a soft, moist, pink layer called the mucosa. The colon begins with the cecum, leading to the right or ascending colon (situated just beneath the liver), followed by the transverse colon, descending colon, sigmoid colon, rectum, and finally the anal canal [6,13,14]. The movement of dosage forms through the colon is slow and can vary significantly, influenced by various factors including diet, fiber intake, physical activity, stress levels, medical conditions, and medications. The gastric emptying of dosage forms varies greatly and is mainly influenced by whether the individual is in a fed or fasted state, as well as the characteristics of the dosage form, including its size and density. The time it takes for an oral dosage form to reach the colon depends on both the rate of gastric emptying and the transit time through the small intestine [6].

Another key factor is the average colonic fluid volume, approximately 13 mL, which varies between 1 and 44 mL in a fasted state. The presence of food alters colonic fluid volume and digestive enzyme levels, impacting nutrient absorption and the digestion of carbohydrates and polysaccharides. In IBD patients, increased fluid secretion and reduced water reabsorption result in a diluted colonic fluid volume. Consequently, reduced colonic fluid volumes can impair drug dissolution and absorption, ultimately affecting the bioavailability of drugs in the colon [15].

The human GIT contains a vast population of both anaerobic and aerobic bacteria. Intestinal enzymes, often sourced from the gut microflora in the colon, are employed to trigger drug release at specific points along the GIT. These enzymes are essential for degrading coatings or matrices and for breaking the bonds between a carrier and the drug, such as in the release of a drug from a prodrug. The bacterial concentration in the human colon is approximately 1000 CFU/mL [6,14].

Antibiotic administration significantly alters the diversity of the gastrointestinal microbiome. By eliminating antibiotic-susceptible bacteria, antibiotics facilitate the proliferation of resistant strains, thereby disrupting microbial equilibrium. Although microbial diversity is reduced, the overall bacterial load in the GIT may increase following antibiotic use. Studies have demonstrated that a seven-day course of β-lactams led to an increased microbial load and an elevated Bacteroidetes-to-Firmicutes ratio [16].

Recent research has highlighted the critical role of the intestinal microbiota in the pathogenesis of IBD. Most studies report reduced microbial diversity in the gut of IBD patients, with notable decreases in Firmicutes and Proteobacteria being key findings [17]. Many human pathogenic bacteria belong to the Proteobacteria phylum, which is increasingly recognized as significant in IBD development. Additionally, an elevated abundance of *Ruminococcus gnavus* has been observed in IBD patients. Consequently, restoring immunological balance by modulating the gut microbiota is now regarded as a promising therapeutic approach for managing IBD [18].

The pH levels in the GIT [depend on factors such as diet, disease, and food intake. Studies using pH-sensitive radio telemetry show that the highest pH level (7.5 ± 0.5) occurs in the terminal ileum, but this drops to 6.4 ± 0.6 upon entering the colon. This decrease in pH is due to the production of short-chain fatty acids resulting from the bacterial fermentation of polysaccharides [12,19]. The consumption of carbohydrate-rich foods can lead to a reduction in colonic pH due to the fermentation of carbohydrates by colonic bacteria, resulting in the production of acidic metabolites [8]. A carbohydrate-rich diet could influence and change the pH levels of the colon. Medications containing polysaccharides can also affect the pH of the colon. For example, colonic bacteria ferment laxatives like lactulose, and the obtained lactic acid reduces the pH of the colon. Some diseases can change pH levels by influencing the microbiota. The pharmacokinetics and pharmacodynamics of a pH-dependent CDDS could be affected by fluctuating pH and by changing the normal conditions of the colon [14,15,16].

The colon is an appropriate site for delivering drugs to treat local colon diseases. Localized treatment requires smaller drug doses, reducing dosage frequency and potentially lowering the cost of expensive medications. This approach may also decrease the likelihood of side effects and drug interactions. Additionally, poorly absorbed drugs may exhibit better bioavailability in the colon while also minimizing gastric irritation, such as that caused by nonsteroidal anti-inflammatory drugs. Drugs can bypass first-pass metabolism here, offering prolonged therapeutic activity during the day or night. The colon’s longer retention time and its responsiveness to absorption-enhancing agents improve patient compliance [20].

## 3. Ulcerative Colitis

Ulcerative colitis is a major form of IBD. UC is a chronic disorder marked by inflammation that typically begins in the rectum and spreads continuously upward, affecting part or all of the colonic mucosa [21]. As a progressive and harmful condition, it can lead to severe complications in the colon. These include persistent diarrhea, the formation of abscesses, the development of fistulas, perforations in the colon wall, and even the risk of neoplasia or colon cancer [3]. The latest guidelines suggest that the management of UC is primarily based on the location of the disease (proctitis, left-sided, or extensive) and its severity. Since UC is a chronic condition with no known preventative or curative treatments, except for colectomy, patients typically require lifelong therapy. The disease naturally alternates between periods of remission and episodes of acute flare-up, which may necessitate intensified treatment, hospitalization, and, in severe cases, surgical removal of the colon [22]. Currently, no medication can cure the condition. During relapses, symptoms such as abdominal pain, diarrhea, and rectal bleeding become more severe. Remission is usually achieved with medical treatment or surgery, though it can sometimes occur spontaneously without intervention [1].

Treatments for UC primarily aim to achieve symptom remission, with the choice of therapy based on the severity of the disease flare-up. In cases of mild-to-moderate UC, the preferred treatment is 5-aminosalicylic acid (5-ASA), also known as mesalamine or mesalazine. However, fast-release oral forms of 5-ASA are often suboptimal, as the drug is absorbed in the upper small intestine and metabolized before reaching effective levels in the inflamed colonic tissues [23]. Also, during remission phases, mesalamine is the drug of choice for treatment.

5-ASA is effective solely as a local treatment, with plasma levels offering no therapeutic benefit. 5-ASA requires delivery directly to the inflamed tissue through targeted-release formulations [23]. Therefore, other 5-ASA-containing aminosalicylates, such as sulfasalazine, olsalazine, and balsalazide, are commonly administered orally to ensure therapeutic effectiveness.

Corticosteroids such as hydrocortisone, budesonide, prednisolone, or beclomethasone are the preferred treatment for inducing remission in patients with moderate-to-severe active UC and for those with mild-to-moderate UC who fail to respond to aminosalicylate therapy. However, due to the significant long-term side effects, including adrenal suppression, immunosuppression, Cushingoid symptoms, and bone resorption associated with corticosteroids such as dexamethasone and methylprednisolone, they are not the preferred treatment options. To address these limitations, specialized colon-targeted formulations could be used [24]. Therefore, targeting drug delivery specifically to the colon could allow for a reduced dose, potentially minimizing these systemic side effects.

Cyclosporine has demonstrated its effectiveness as a rescue therapy for severe UC. However, it should not be used for maintenance treatment but rather as a transitional option leading to other therapies, such as thiopurines, biologics, or surgery [25].

Biotechnological drugs and small molecules like anti-tumor necrosis factor agents (infliximab, adalimumab, golimumab, and certolizumab) or anti-integrin agents are also gaining attention, as a growing range of them are now available for treatment [26]. The primary benefit of biological agents is their capacity to sustain remission and promote mucosal healing without the need for corticosteroids. However, these biological drugs, along with other immunosuppressants, can also have significant side effects, as they impair the immune system’s ability to combat infections, raising the risk of serious viral, bacterial, or fungal infections that could become systemic.

Unlike small molecules, biologics are expensive to manufacture, have molecular weights exceeding 1000 kDa requiring parenteral administration, possess long half-lives, and exhibit antigenic properties that reduce their long-term effectiveness [27]. Janus kinase inhibitors represent the first class of innovative small molecules approved in multiple countries for managing IBD. The combination of different classes of therapeutic agents has also been investigated. Interest is increasing in combining biologic agents with small molecules as a therapeutic strategy. This approach is based on the idea that targeting distinct and potentially complementary pathways could improve efficacy while reducing side effects. Data from randomized controlled trials show that combining infliximab with immunomodulators is more effective in achieving corticosteroid-free remission. Treatment for UC is advancing quickly, with a rising number of medications and therapeutic strategies under development and evaluation. Soon, clinicians will have access to a broader array of agents with varied mechanisms of action to manage UC effectively [27,28].

Colon-specific drug delivery systems (CDDSs) offer an optimal therapeutic approach to enhance dosing effectiveness, maximize therapeutic benefits, and minimize adverse effect [23].

## 4. Colon-Specific Drug Delivery Systems

Effective colonic drug delivery requires thoughtful consideration of the drug’s characteristics, the delivery system used, and how it interacts with either a healthy or diseased gut. The drug must first dissolve in the fluid present in the colon. However, the colon has less free fluid compared to the small intestine, which can pose challenges for drugs with poor water solubility [6]. To address this, the drug may need to be administered in a pre-solubilized form or targeted specifically to the proximal colon [1].

**Figure 1:**
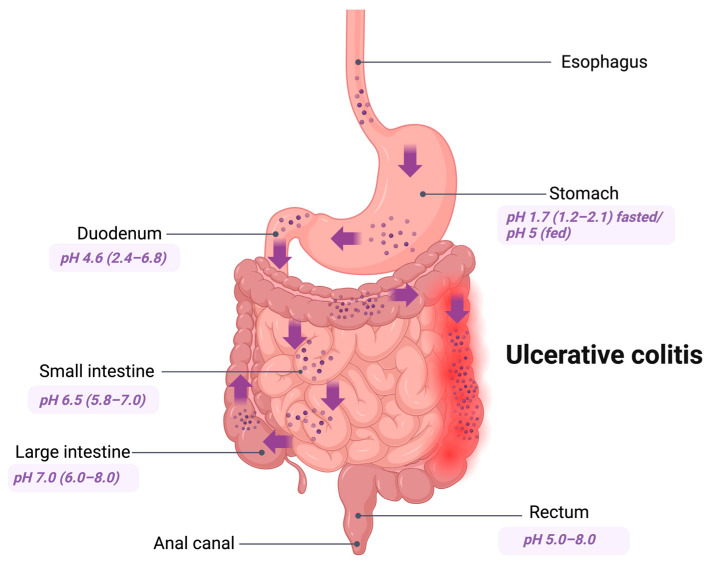
Colon-targeted drug delivery to the UC area. Created at https://BioRender.com (accessed on 20 December 2024). Note: The arrows indicate the transition of particulate colon-specific drug delivery systems to the target site affected by UC; The circles represent particulate CDDS.

At present, innovative oral CDDSs have emerged as a type of site-specific drug delivery method designed to target the colon for the administration of various medications [29] (Figure 1). An ideal drug delivery system like a CDDS should ensure that the entire drug reaches the colon, with minimal delivery to the upper GIT. This site-specific approach ensures the maximum therapeutic effect [30]. By specifically delivering drugs to the colon, CDDSs offer significant advantages, such as enhancing safety and reducing toxicity in the treatment of local or systemic chronic diseases like UC [31].

Colon drug targeting can be achieved through several techniques, including prodrug formation, the use of pH-sensitive or biodegradable polymer coatings, formulations based on polysaccharides, time-dependent systems, pressure-controlled drug delivery, osmotic pressure-controlled systems, and delivery methods that utilize colon-resident bacteria or bacteria-produced enzymes to trigger drug release [32].

Enteric coatings are widely recognized, and many marketed UC treatments utilize them to delay drug release, aiming to enhance local drug delivery. These coatings have shown benefits for IBD patients. However, the pH profiles in the GITs of individuals with active IBD indicate that precise control over the release site—especially from the terminal ileum onward—is challenging when relying solely on pH-sensitive polymer coatings. Effective UC treatment can be achieved by orally delivering drugs through colon-specific systems, allowing the medication to act both locally and systemically [23].

The limitations of CDDSs include the need for multiple manufacturing steps and the potential impact of resident microflora, which can metabolize and degrade the drug. Drug release may be incomplete, and bioavailability can be reduced due to non-specific binding to mucus, fecal matter, dietary residues, and intestinal secretions. Additionally, drugs must be in solution form for absorption, making dissolution a limiting factor for drugs with poor solubility [33]. There is also a lack of appropriate in vitro dissolution testing methods to evaluate dosage forms effectively. The prodrug approach has limitations as well, as it relies on the availability of specific functional groups on the drug for chemical linkage, making it less versatile. Furthermore, prodrugs are considered new chemical entities and require extensive evaluation before use as carriers [20].

Time-dependent drug delivery systems can be developed by coating drug cores to delay release through various mechanisms, using osmotic devices, or by designing dosage forms with specific shapes, such as capsules. However, a limitation of these systems is the lack of precise targeting, as variations in gastric emptying times can affect the timing of drug release [29,32]. Various factors, such as the presence of disease, surgery, concurrent medications, or dietary changes, can affect patients’ gastrointestinal transit times, causing time-based drug delivery methods to fail in consistently targeting the colon [31].

Microbially triggered drug delivery is based on the principle that colonic microflora degrades polymers coated on the dosage form. The colon hosts a diverse range of microflora responsible for fermenting substrates like polysaccharides present in the intestinal tract. These microflora produce various enzymes that metabolize polysaccharides, carbohydrates, and proteins, thereby bypassing digestion in the upper GIT [33,34].

Polymers susceptible to microbial degradation include pectin, dextran, chitosan, cyclodextrin, alginates, amylose, and locust bean gum. However, the effectiveness of microbial-triggered drug delivery can be limited when the colonic microflora is disrupted, such as during treatment with antibiotics or chemotherapeutic agents [35]. Imbalances in the gut microbiota have also been associated with several diseases, including inflammatory bowel disease, obesity, diabetes, and even mental health disorders [11]. That is why this approach also has limitations in treating UC.

The effectiveness of oral colon-targeted drug delivery systems relies on addressing various physiological barriers within the gastrointestinal tract, including variable transit times, pH levels, fluid volumes, enzymatic activity, microbiota diversity, and the mucus layer. Additionally, factors such as the presence of food and individual variability also play a significant role in influencing drug delivery to the colon and should be carefully considered during the design of such systems [15].

### Dosage Forms Suitable for Targeted Delivery

Multiparticulate systems are among the most effective candidates for obtaining a CDDS. These systems offer several advantages, including reduced local irritation and systemic toxicity and enhanced bioavailability. The techniques used in multiparticulate systems include pellet formation, microparticle preparation, granule production, and nanoparticle formulation [35].

Multiparticulate approaches are preferred over single-unit dosage forms like tablets because they allow the drug to reach the colon more quickly and remain there for an extended period. Due to their small and fine size, multiparticulate systems can easily pass through the GIT. Additionally, these systems are more uniformly dispersed in the GIT than single-unit systems, leading to improved drug absorption [36].

Nanoparticle preparation is a straightforward process. This formulation offers high stability by protecting protein and peptide drugs from chemical and enzymatic degradation in the GIT, thereby enhancing their absorption through the intestinal epithelium. Various techniques are used to produce polymeric nanoparticles, including polymerization, nanoprecipitation, and inverse microemulsion, which involve the application of agitation, organic solvents, and heat. However, the use of heat and agitation pose a significant limitation, as these conditions can be detrimental to protein and peptide drugs [34].

Microparticles are a preferred choice for colon targeting due to their versatility in preparation and ease of coating with pH-sensitive polymers. The mechanism of drug delivery to the colon using microparticles relies on variations in pH and gastrointestinal transit [36].

Compared to single-unit dosage forms such as tablets and capsules, multiparticulatedrug delivery systems have demonstrated superior performance for colon targeting. These systems facilitate faster drug delivery to the colon and allow the drug to remain in the ascending colon for an extended duration. Their smaller particle size enables them to traverse the GIT more easily, resulting in reduced inter- and intrasubject variability. Additionally, multiparticulate systems exhibit more uniform dispersion and absorption compared to single-unit dosage forms [35].

The use of multiparticulates can improve patient compliance while preserving the desired in vivo drug absorption profile. Typically, formulations that are simple, easy to manufacture, and scalable using well-established techniques are preferred to ensure product consistency and reproducibility [37].

The use of microscopic particles, such as micro- and nanoparticles, in targeted drug delivery systems introduces additional production challenges due to the structural complexity of these systems. It is essential to precisely control various structural parameters, including particle size, size distribution, surface morphology, surface chemistry, porosity, pore size distribution, pore connectivity, drug loading capacity, and the spatial distribution of the drug within the particles, all in a consistent and reproducible manner. Moreover, achieving this level of control is necessary not only for laboratory-scale production but also for industrial-scale manufacturing. The production process must be easily capable of changes in scale, particularly scaling up [38].

## 5. pH-Dependent Drug Delivery Systems

One pH-sensitive drug delivery system is a polyelectrolyte containing ionizable groups within its main chain, side groups, or terminal groups. Variations in pH trigger these carriers to alter their structure, such as swelling or collapsing, dissolving or precipitating, micellizing or demicellizing, undergoing conformational changes, and modifying their hydrophilic or hydrophobic surfaces [39]. Polymers that respond to pH are gaining popularity as efficient carriers for delivering therapeutic molecules, including drugs, nucleic acids, and proteins [40].

In this approach, the difference in pH between the upper and lower sections of the GIT is utilized to efficiently target drug delivery to the colon. The pH levels in the GIT are known to range from 1.2 in the stomach to between 7.5 and 8 in the large intestine. As a result, pH-sensitive polymers become the initial approach for targeted drug delivery to the colon.

Designing a delivery system that can reliably handle the pH variability encountered as it moves from the stomach to the small intestine is a major challenge. By leveraging insights into polymer properties and their solubility across different pH levels, systems have been developed to release drugs specifically at the desired target site [39]. Key factors influencing the effectiveness of these systems include the combination of various polymers, pH of the surrounding medium, tablet coating thickness, and the presence of plasticizers. Additional variables, such as inter- and intra-patient differences, electrolyte levels, and transit time, also play crucial roles in the system’s success. Despite these challenges, pH-sensitive systems are available commercially, including mesalazine-based products (Asacol^®^ with a Methacrylsäure–Methylmethacrylat copolymer (1:2) (Ph.Eur.) and Salofalk^®^ with Eudragit^®^ L) and budesonide products (Budenofalk^®^ with a Methacrylsäure–Methylmethacrylat copolymer (1:1) (Ph.Eur.), a Methacrylsäure–Methylmethacrylat copolymer (1:2) (Ph.Eur.), and Entocort^®^ with a Methacrylsäure–Ethylacrylat copolymer (1:1) (Ph.Eur.) for UC treatment [12].

### 5.1. Advantages of pH-Dependent Drug Delivery Systems

There are numerous approaches (microencapsulation, nanoencapsulation, or just the coating of drug microparticles or the drug core) to achieve pH-dependent delivery systems using pH-sensitive polymers. Most of them are widely available and cheap. Additionally, combining different polymers can improve the efficiency of pH-sensitive drug delivery systems or pH-dependent drug release mechanisms [41]. The drug is directly targeted to a specific site of the colon, which decreases the dose to be administered and the possible side effects. Drug loss can be minimized by avoiding extensive first-pass metabolism. The pH-dependent delivery systems allow the protection of the gastric and small intestine mucosa from irritating drugs [42]. This is because the dosage form is preserved intact when passing through the stomach and small intestine, or only the acid-labile layer is released.

pH-responsive drug delivery systems can enhance the bioavailability of the drug and enable its sustained, targeted delivery. They effectively minimize the adverse effects of the drug and the surrounding conditions. By encapsulating the drug within polymer systems, these systems protect the drug from degradation or inactivation due to the biological environment while also reducing the drug’s side effects on healthy tissues [40].

### 5.2. Disadvantages of pH-Dependent Drug Delivery Systems

One drawback of the pH-sensitive approach is the unpredictable fluctuations in gastrointestinal pH in some diseases. The pH in the gastrointestinal (GI) tract can be significantly influenced by various factors, including diet, illnesses, hydration levels, and the metabolic activities of gut microbes. Additionally, the influence of whether the patient is fasted or fed can affect pH levels and affect the effectiveness of this approach [23]. Consequently, both internal and external factors can disrupt pH levels, potentially compromising the effectiveness of pH-dependent drug delivery systems and resulting in suboptimal site-selective drug release [43]. pH-sensitive systems tend to release their contents rapidly as soon as a change in pH is detected.

pH-dependent drug delivery could be achieved by coating the drug core with pH-sensitive polymers (enteric coatings), pH-responsive hydrogels, pH-triggered microparticles/nanoparticles, pH-responsive polymeric prodrugs, and other coatings [11,44,45,46].

Enteric coatings using pH-sensitive polymers are engineered to stay intact in the stomach’s acidic environment but break down or dissolve in the more alkaline pH of the small intestine. This protective layer prevents the drug from being exposed to gastric acid, ensuring it reaches the small intestine’s absorption site without premature release or degradation [11,45,47].

pH-triggered microparticles and nanoparticles are composed of materials and polymers that respond to changes in pH, allowing for controlled drug release. These systems are designed to remain stable in one pH environment and then release the drug when they reach a different pH level [11,46].

Hydrogels are three-dimensional networks of hydrophilic polymers capable of absorbing large amounts of water. pH-responsive hydrogels are designed with polymers that undergo swelling or dissolution in response to changes in pH. When drugs are incorporated into these hydrogels, such as in microbeads, they can serve as effective pH-dependent drug delivery systems [47]. These hydrogel microbeads remain intact in the acidic environment of the stomach but swell and release the drug when exposed to the more neutral pH of the intestines [11,46].

pH-responsive polymeric prodrugs are inactive drug conjugates that undergo chemical changes when exposed to specific pH conditions. These prodrugs often contain linkers that can be cleaved under certain pH conditions, thereby releasing the active drug. This approach provides a strategy for site-specific drug delivery and controlled release, taking advantage of the pH characteristics of the target tissue or organ [11,44].

The versatility of these systems makes them valuable for a wide range of applications, including oral drug delivery, colon-specific treatments, and cancer therapy. Enteric coatings are ideal for protecting acid-labile drugs, while hydrogels provide flexibility in achieving controlled release. Microparticles and nanoparticles ensure efficient delivery and absorption due to their small size and responsive properties. Polymeric prodrugs offer an advanced solution for site-specific targeting based on environmental triggers [11,46,47].

pH-responsive antibodies: Antibodies can be designed with the capability to respond to a wide range of environmental and physiological triggers within their cellular surroundings, including pH changes. This characteristic is crucial for enhancing the therapeutic efficacy of conventional antibodies. As a result, the development of pH-responsive antibodies is rapidly gaining attention. The pH spectrum within the human body offers an opportunity for the development of pH-responsive antibodies. The effective pH range for these antibodies to serve as therapeutics includes the acidic tumor microenvironment (pH 5.9) and neutral human plasma (pH 7.4) [48]. Currently, such antibodies are being developed for use in the therapy of tumors and neurodegenerative diseases. pH-sensitive antibodies hold significant therapeutic promise. These adaptable antibody-based therapies have the potential to become a valuable asset in a physician’s toolkit for treating UC.

Despite their advantages, challenges such as scalability, reproducibility, and stability during manufacturing need to be addressed to optimize these systems for industrial applications. Overall, pH-dependent drug delivery systems represent a promising strategy to enhance drug efficacy, reduce side effects, and achieve site-specific targeting, significantly improving therapeutic outcomes [38].

## 6. pH-Dependent Polymers

**Figure 2:**
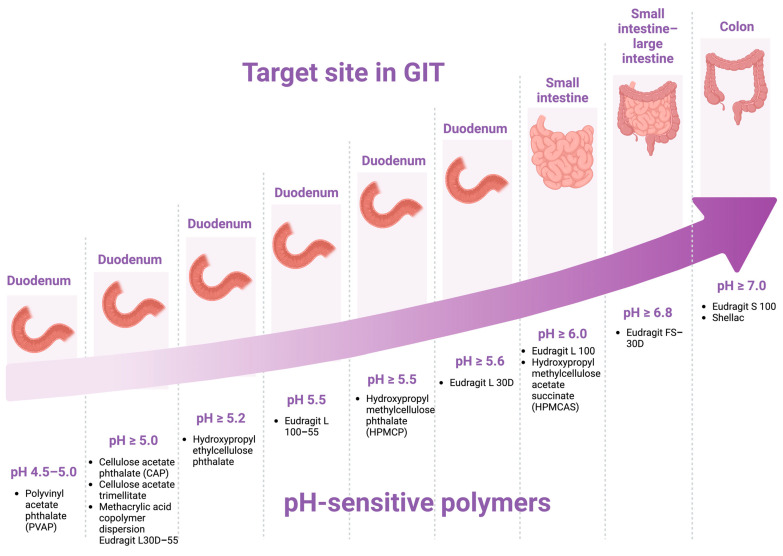
pH-dependent polymers and their pH-threshold of dissolution. Created in https://BioRender.com (accessed on 20 December 2024).

The optimal pH-sensitive polymer should resist degradation in the acidic environment of the stomach and the upper small intestine but dissolve at the higher pH levels found in the terminal ileum and colon. Consequently, drug delivery systems coated with pH-sensitive polymers that dissolve within a pH range of 6.0–7.0 (Figure 2) are designed to delay drug release and prevent premature dissolution in the upper GIT, ensuring targeted delivery to the colonic regions [49].

pH-responsive polymers are composed of acidic or basic groups capable of donating or accepting protons in reaction to changes in the surrounding pH. They react to variations in the surrounding pH by exhibiting changes in their structure and properties, including surface activity, chain conformation, and solubility. The specific nature of these changes is determined by the structural characteristics of the pH-sensitive material [50]. It is important to note that in the treatment of IBD the use of anionic polymers can be advantageous, as they bind to the positively charged proteins present in inflamed tissues [15].

pH-dependent polymers can be categorized into two types based on their origin: natural and synthetic.

Natural pH-responsive polymers can be differentiated from synthetic ones based on their origin. Biopolymers often exhibit pH-responsive conformational changes accompanied by shifts in solubility [51]. Natural polymers are widely utilized due to their abundance in nature, biodegradability, non-toxic properties, biocompatibility, and ease of modification. Examples of such natural PRPs include hyaluronic acid, alginic acid, heparin, chitosan, and cellulose derivatives like carboxymethyl cellulose and carboxymethyl dextran [52].

While natural polymers are widely favored for their advantages, synthetic polymers like polypeptide derivatives, including poly(L-glutamic acid), poly(histidine), and poly(aspartic acid), offer biocompatibility and degradability too, alongside their pH-sensitive properties [53]. In biological systems, cellular functions are controlled by biomacromolecules that respond to changes in the local physiological environment [54]. Synthetic polymers serve as effective biomimetic tools, as they can adapt to and interact with complex biological environments [55].

Methacrylic acid and methyl methacrylate are the most commonly used synthetic pH-responsive polymers for colonic drug delivery. Such are the polymers of the Eudragit^®^ series by Evonik Industries. The ratio of ester to carboxylic acid functional groups differs among various Eudragit^®^ derivatives, influencing their solubility at specific pH levels [56]. Drug release from these pH-sensitive polymers occurs through a mechanism where the carboxylic acid groups deprotonate upon reaching the target pH range (6.8–7.4). This triggers polymer swelling, followed by processes such as polymer erosion, dissolution, or a combination of these mechanisms, ultimately causing a rapid burst release of the drug into the surrounding medium [57].

The methacrylic acid copolymers, such as methacrylic acid–methyl methacrylate copolymer (1:1) Eudragit^®^ L100 and methacrylic acid–methyl methacrylate copolymer (1:2) Eudragit^®^ S100, have commonly been used as pH-dependent coating agents aimed at protecting the drug core from the severe gastric environment [58]. Eudragit^®^ S100, a widely studied biocompatible polymer for colon-targeted drug delivery, has been approved for oral use by regulatory agencies in the U.S., Europe, and Japan [59]. This polymer dissolves selectively in aqueous environments with a pH of 6–7, enabling the release of the encapsulated drug specifically in the colon [60].

## 7. Historical Overview of pH-Dependent Strategies Utilizing Various Substances for UC Treatment over the Past 15 Years 

### 7.1. Metronidazole

A research team of Vaidya et al. [61] developed a multiparticulate system featuring pH-sensitive properties and enzyme-specific biodegradability for the colon-targeted delivery of metronidazole (Table 1). Pectin microspheres were fabricated using an emulsion dehydration method and subsequently coated with Eudragit^®^ S100 via an oil-in-oil solvent evaporation technique. In vitro drug release studies demonstrated that the formulation exhibited no release at gastric pH but showed sustained drug release at colonic pH. In vivo studies further validated the system’s effectiveness by analyzing drug concentrations across different regions of the GIT over time, confirming its potential for colon-specific drug delivery. This evidence supports the use of Eudragit^®^-coated pectin microspheres as an effective approach for targeting drugs to the colon [61].

### 7.2. Budesonide

Makhlof et al. [62] developed pH-sensitive nanospheres (Table 1) using a polymeric blend of poly(lactic-co-glycolic) acid and a pH-sensitive methacrylate copolymer. Budesonide, a topically active corticosteroid, was encapsulated as a model drug. The therapeutic efficacy of these nanospheres was evaluated using a trinitro benzenesulfonic acid colitis rat model compared to conventional enteric microparticles. The nanospheres exhibited strongly pH-dependent drug release, with minimal release at acidic and neutral pH levels, followed by a sustained-release phase at pH 7.4. In animal studies, budesonide-loaded nanospheres demonstrated superior therapeutic effectiveness in mitigating symptoms of induced colitis. Furthermore, quantitative fluorescent marker analysis and confocal laser scanning microscopy revealed the strong and targeted adhesion of the nanospheres to the ulcerated and inflamed mucosal tissue in the rat colon. In summary, the developed nanosphere system integrates pH sensitivity, controlled drug release, and particulate targeting, presenting a promising approach for colon-specific drug delivery in IBD such as UC [62].

Yoo et al. [63] developed budesonide-loaded nanoparticles (Table 1) with dual pH- and time-sensitive properties for colitis treatment. These nanoparticles were formulated using Eudragit^®^ FS30D as a pH-responsive polymer and Eudragit^®^ RS100 for time-controlled drug release. Produced through the oil-in-water emulsion technique, the pH/time-sensitive nanoparticles were specifically engineered to avoid premature drug release in acidic conditions while ensuring sustained release at colonic pH levels. The therapeutic potential and distribution of these nanoparticles were evaluated in a dextran sulfate sodium-induced colitis mouse model. The pH/time-sensitive nanoparticles effectively minimized burst release in acidic environments and delivered budesonide directly to the inflamed colon. Mice treated with these nanoparticles demonstrated significant improvements, including reduced disease activity index scores, better colon weight-to-length ratios, less histological damage, and decreased inflammatory cell infiltration. These results underscore the potential of dual pH/time-sensitive nanoparticles as a promising oral drug delivery system for targeted colitis treatment [63].

Naeem et al. [64] developed an innovative hybrid drug delivery system for budesonide (Table 1), combining nanoparticles and microparticles for the targeted treatment of colitis. The system involved first formulating sustained-release poly(lactic-co-glycolic acid) nanoparticles, which were then encapsulated within pH-sensitive Eudragit^®^ FS30D microparticles. This dual-encapsulation strategy provided robust protection for the drug in gastric and intestinal-like pH conditions while enabling selective release in the colon. In vitro studies demonstrated that the obtained hybrid drug delivery system effectively inhibited drug release at acidic and intestinal pH levels, transitioning to a controlled and sustained release at ileal and colonic pH due to the degradation of the pH-sensitive outer microparticles. In vivo imaging of colitis-induced mice orally administered a fluorescent hybrid drug delivery system showed intense fluorescence localized in the colon, confirming the release of nanoparticles and their accumulation at inflamed sites. Compared to treatment with nanoparticles alone, the obtained hybrid formulation significantly alleviated the symptoms of experimental UC in mice. This research highlights a promising platform for enhancing nanoparticle-based colon-targeted drug delivery by providing improved protection and controlled release of therapeutic agents in the GIT [64].

Turanli et al. [65] designed budesonide-loaded nanoparticles (Table 1) with both pH- and time-dependent release properties for the treatment of IBD. Anionic polymethacrylate (Eudragit^®^ S100) served as the pH-sensitive polymer, while cationic polymethacrylate (Eudragit^®^ RL100) was used for sustained, time-dependent release. The nanoparticles were prepared using a single oil-in-water emulsion/solvent evaporation method. A formulation with a 90% anionic to 10% cationic polymer ratio successfully minimized burst drug release under acidic conditions and provided sustained drug release at a neutral pH. These findings indicate that budesonide-loaded nanoparticles with combined pH- and time-dependent properties represent a promising drug delivery system for effective IBD management [65].

### 7.3. Prednisolone

Alkazzaz et al. [66] developed colon-targeted, enteric-coated tablets containing various prednisolone solid dispersion formulations (Table 1) to treat UC, enhance patient compliance, and minimize gastrointestinal side effects. They prepared prednisolone solid dispersions using D-mannitol, polyethylene glycol 4000, and Kollicoat IR to deliver the drug to the colon in a pre-solubilized form. Then, the optimized formulation was compressed into fast-disintegrating tablets and coated with Eudragit^®^ S100. The study explored different factors like carrier type, drug-to-carrier ratio, and coating thickness to assess their impact on drug solubility and dissolution. The study showed that 1:3 prednisolone/Kollicoat IR solid dispersions demonstrated the best dissolution rate improvement. The 16% coating level (Eudragit^®^ S100, dibutyl phthalate, and talc) provided an ideal lag time of 5 h, resisting pre-colonic pH and ensuring immediate release in pH 7.4. This study offers a promising colonic delivery system for prednisolone with enhanced solubility and bioavailability [66].

Liu et al. [67] introduced an innovative double-coating strategy (Table 1) using the acrylic polymer Eudragit^®^ S to enhance ileocolonic drug targeting. This system features an inner layer of partially neutralized Eudragit^®^ S combined with a buffering agent, covered by an outer layer of standard Eudragit^®^ S. Tablets containing prednisolone were coated with double-layer formulations of varying inner coat compositions, and a conventional single-coating method was used for comparison. Drug release was evaluated using the USP II apparatus, first in 0.1 M HCl for 2 h, followed by pH 7.4 Krebs buffer, a medium mimicking the ionic properties and buffering capacity of the distal small intestine. The results indicated that drug release from the single-layer-coated tablets in the pH 7.4 Krebs buffer was delayed by 120 min, whereas the double-coated tablets released the drug significantly faster. The release profile was influenced by the pH and buffer capacity of the inner coating. The optimal double-coating formulation, with an inner layer containing 10% KH_2_PO_4_ (neutralization pH of 8.0), achieved a drug release lag time of 40 min. The enhanced dissolution of the double coating and the subsequent rapid drug release demonstrate its potential as an effective targeting system [67].

Hashem et al. [68] employed the solvent evaporation method to prepare prednisolone microspheres (Table 1) using various ratios of ethyl cellulose and Eudragit^®^ S100, with 0.5% and 1% *w*/*v* Span^®^ 80 as an emulsifier. The anti-inflammatory activity of a selected microsphere formulation was compared to that of conventional prednisolone tablets. The findings revealed that the optimal microsphere formulation demonstrated the ability to specifically deliver prednisolone to the colon, potentially reducing dosing frequency and minimizing systemic side effects associated with conventional prednisolone tablets. The study highlights promising results regarding the therapeutic effectiveness of colon-targeted microspheres designed with a combination of two different polymers [68].

### 7.4. Budesonide and Prednisolone

Qu et al. [69] developed and optimized a colon-targeted oral drug delivery system (Table 1) utilizing mesoporous silica nanoparticles coated with Eudragit^®^ S100. The drug encapsulation and pH-responsive polymer coating were accomplished using a one-step rotary evaporation method. Corticosteroid drugs, including prednisolone (16.8% *w*/*w*) and budesonide (9.0% *w*/*w*), were successfully encapsulated and protected by the Eudragit^®^ S100 coating. The coating effectively shielded the drugs from the acidic environment of the GIT, preventing premature release before reaching the colonic site. Encapsulation of budesonide within mesoporous silica nanoparticles significantly enhanced its anti-inflammatory effects, particularly in the distal colon, compared to the free drug. This study highlights the potential of pH-responsive mesoporous silica nanoparticles as a cost-efficient and effective oral drug delivery system for improved IBD treatment [69].

### 7.5. Hydrocortisone Sodium Succinate (HSS)

To mitigate the adverse effects of corticosteroid therapy in UC, Shi et al. [70] developed pH-sensitive hydrogel microspheres (Table 1) loaded with HSS, an anti-inflammatory drug known for its significant side effects. This type of hydrogel microsphere was obtained by the emulsion crosslinking method. The microspheres were prepared using itaconic acid, an amphiphilic polymer (created via a ring-opening reaction between polyethylene glycol methyl ether and polylactic acid), and poly(ethylene glycol) methyl ether methacrylate. Bisacrylamide served as the crosslinking agent, while ammonium peroxydisulfate acted as the polymerization initiator in the synthesis process. In vitro studies of the HSS-loaded hydrogel microspheres demonstrated desirable pH sensitivity, with a cumulative release rate of 4.07% at pH 1.2 and 94.64% at pH 7.4 over 12 h. This indicated that the HSS-hydrogel microspheres could effectively deliver HSS to the colon while reducing premature absorption in the upper GIT. In experimental colitis models in mice, the HSS-HMSs exhibited superior therapeutic and ameliorative effects compared to HSS alone. The researchers concluded that the HSS-hydrogel microspheres had significant potential as a targeted drug delivery system for the treatment of UC [70].

### 7.6. Dexamethasone

Oshi et al. [71] developed colon-targeted dexamethasone microcrystals (Table 1) coated with sequential layers of chitosan oligosaccharide, alginate, and Eudragit^®^ S100 using a layer-by-layer coating technique. These microcrystals exhibited pH-sensitive drug release, effectively preventing an initial burst release in the acidic environments of the stomach and small intestine while ensuring sustained release at colonic pH. Notably, the optimized formulation demonstrated significantly improved therapeutic efficacy in a mouse model of colitis compared to other dexamethasone microcrystal variants. This study highlights the potential of layer-by-layer-coated dexamethasone microcrystals as an effective colon-targeted treatment for IBD [71].

### 7.7. Coumarin

Naeem et al. [72] developed nanoparticles (Table 1) with dual sensitivity to enzymes and pH, specifically designed for colon-targeted drug delivery. The nanoparticles utilized a polymeric blend of enzyme-responsive azo-polyurethane and pH-sensitive Eudragit^®^ S100. This dual-sensitive system aimed to optimize drug release for treating inflamed colon regions while minimizing premature drug release in the stomach and small intestine. The researchers fabricated both single-pH-sensitive and dual-sensitive nanoparticles using an oil-in-water emulsion solvent evaporation technique, incorporating coumarin-6 as a model drug. The single-pH-sensitive nanoparticles exhibited an almost complete burst release at pH 7.4, while the dual-sensitive nanoparticles demonstrated the effective prevention of burst release, followed by a controlled, sustained drug release phase. In vivo experiments in rat GITs revealed that the dual-sensitive nanoparticles achieved selective accumulation in the inflamed colon, with coumarin-6 concentrations 5.5 times higher than those delivered by single-pH-sensitive nanoparticles. These findings highlight the potential of enzyme/pH dual-sensitive nanoparticles as a robust approach for colon-specific drug delivery, particularly for conditions like IBD and other colon-related disorders [72].

### 7.8. Notoginsenoside

Tie et al. [73] conducted a study to develop a pH-dependent solid dispersion system for the oral colon-targeted delivery of notoginsenoside R1(Table 1). Although research indicates that notoginsenoside R1 possesses significant anti-inflammatory properties, its clinical application is hindered by challenges such as acid-induced degradation and poor bioavailability. The formulation utilized Eudragit^®^ S100 and polyethylene glycol 4000 as pH-sensitive carriers, with R1 solid dispersions prepared via the solvent evaporation method. In vitro release studies of the optimized notoginsenoside R1 solid dispersions showed no drug release in gastric-like conditions (pH 1.2). However, significant release occurred under intestinal conditions, with less than 30% release in pH 6.8 phosphate-buffered saline and more than 90% release in pH 7.6 conditions. An in vitro colon absorption test demonstrated that the notoginsenoside R1 solid dispersions achieved higher absorption and cumulative release rates compared to notoginsenoside R1 alone. In vivo studies in mice with UC revealed that the notoginsenoside R1 solid dispersions effectively mitigated symptoms such as colon shortening, inflammatory tissue damage, weight loss, diarrhea, and bloody stool. The protective effects of the formulation were superior to those of notoginsenoside R1, highlighting its potential for practical application in managing IBD [73].

### 7.9. Cyclosporine

Cyclosporine A (CsA), a powerful immunosuppressant, is effective in managing UC, but its clinical application is hindered by severe systemic side effects. To overcome this limitation, Oshi et al. [74] developed a colon-targeted drug delivery system (Table 1) using CsA crystals encapsulated within alginate microparticles coated with Eudragit^®^ S100 (CsAc-EAMPs). This delivery system was designed to reduce systemic exposure while enhancing therapeutic efficacy. CsA crystals were synthesized through anti-solvent precipitation, followed by encapsulation via ionic gelation and coating with Eudragit^®^ S100. In vitro studies showed that CsA release from the obtained CsA crystals was effectively suppressed in simulated gastric and intestinal environments, minimizing systemic absorption and related side effects. Upon reaching simulated colonic conditions, the Eudragit^®^ S100 coating dissolved, the alginate microparticles disintegrated, and CsA was released in a sustained manner over 24 h. In a dextran sulfate sodium-induced colitis mouse model, the CsA crystals enabled effective colonic delivery and significantly enhanced the anti-inflammatory effects. These findings suggest that the obtained CsA crystals represent a promising approach for targeted UC therapy [74].

### 7.10. Iridoid Glycosides

Iridoid glycosides, the primary active components of Syringa oblata Lindl., exhibit proven anti-inflammatory effects for treating UC. However, their commercial use is hindered by poor bioavailability and an inability to effectively reach inflamed colon tissue. To address these limitations, Gao et al. [75] developed dual-functional iridoid glycoside-loaded nanoparticles (Table 1) designed to enhance colonic residence time. The nanoparticles were formulated using the oil-in-water emulsion technique. Poly(lactic-co-glycollic acid) was chosen as a sustained-release polymer, while Eudragit^®^ S100 and Eudragit^®^ L30D-55 were incorporated as pH-responsive polymers. The therapeutic efficacy and gastrointestinal transport of the obtained nanoparticles were evaluated using a rat colitis model. The nanoparticulate system demonstrated significantly higher accumulation in the colon, indicating effective targeting facilitated by the pH- and time-responsive properties of the polymers. Gao et al. confirmed that these functionalized nanoparticles containing iridoid glycosides could markedly improve colonic damage, highlighting their potential for UC treatment.

### 7.11. Flurbiprofen

Flurbiprofen, a nonsteroidal anti-inflammatory drug, is used to manage UC. However, its short biological half-life necessitates frequent dosing, which can lead to adverse effects such as gastric ulceration and bleeding. To address these challenges and enhance patient compliance, Pande et al. [76] developed ileocolonic-targeted mucoadhesive microspheres for Flurbiprofen delivery (Table 1). The core microspheres were prepared with chitosan using the emulsification crosslinking method. To ensure colon-specific delivery, the microspheres were coated with enteric polymers, Eudragit^®^ L 100 and Eudragit^®^ S 100, using the emulsion solvent evaporation technique. In vitro release studies revealed that microspheres coated with a combination of Eudragit^®^ L 100 and Eudragit^®^ S demonstrated sustained and targeted drug release profiles. The chitosan-based microspheres with enteric coatings hold promise as an effective carrier system for the colon-specific delivery of Flurbiprofen, potentially improving therapeutic outcomes and reducing gastric side effects in UC treatment [76].

### 7.12. Baicalin

Huang et al. [77] developed colon-targeting Baicalin (BA) granules using plasticizer dry powder coating technology to enhance the targeted delivery of BA (Table 1). BA, a flavonoid derived from the dried root of *Scutellaria baicalensis Georgi*, exhibits potent anti-inflammatory properties, making it a promising candidate for treating UC. Eudragit^®^ S100 was employed as the polymer for colon-specific drug delivery. In vivo animal imaging studies demonstrated that the granules effectively transported BA to the colon while inhibiting its release in the upper GIT. Therapeutic evaluation in colitis models showed that the granules significantly reduced tumor necrosis factor-alpha and interleukin-1β expression levels while increasing superoxide dismutase activity in colonic tissues. Huang et al. concluded that Eudragit^®^ S100 is appropriate for creating multi-unit oral colon-targeted formulations using plasticizer dry powder coating. The resulting granules exhibited excellent colon-targeting properties, enhancing therapeutic efficacy and presenting a promising approach for UC management [77].

### 7.13. Methotrexate

Lv et al. [78] aimed to mitigate the side effects of methotrexate (MTX), an immunosuppressant used for UC treatment, by developing MTX-loaded nanoparticles (Table 1). These nanoparticles were formulated using polylactic-co-glycolic acid, Eudragit^®^ S100, chitosan, and hyaluronic acid. In vitro drug release studies under simulated gastrointestinal conditions revealed that the nanoparticles exhibited pH-sensitive properties, enabling targeted drug release in the colon. Enhanced cellular uptake of the nanoparticles was observed in macrophages, resulting in inhibited proliferation and the reduced secretion of pro-inflammatory cytokines. In vivo imaging further confirmed that the MTX nanoparticles accumulated specifically in the colon of colitis-induced mice and demonstrated a prolonged retention time at the site of inflammation. These findings highlight the potential of MTX nanoparticles with colon-specific and macrophage-targeting capabilities as an effective nanotherapeutic strategy for IBD treatment [78].

### 7.14. Curcumin and Combinations

Gugulothu et al. [79] developed pH-sensitive nanoparticles (Table 1) encapsulating a combination of celecoxib, a selective cyclooxygenase-2 inhibitor, and curcumin, a natural antioxidant and anti-inflammatory agent, to treat UC. Both drugs have been shown to alleviate UC, and their combination was encapsulated using Eudragit^®^ S100 via an emulsion solvent evaporation technique. The synergistic action of the drug combination, along with the advantages of nanosized carriers and the pH-sensitive polymer, allowed for reduced overall toxicity, a lowered celecoxib dosage, and enhanced therapeutic efficacy. This hypothesis was validated in a rat model of UC, where the pH-sensitive nanoparticles of the drug combination demonstrated superior efficacy compared to nanoparticles containing either the drugs alone or their suspension [79].

Xiao et al. [60] utilized the emulsion solvent evaporation technique to fabricate curcumin microparticles (Table 1) using pH-sensitive Eudragit^®^ S100 and poly(lactide-co-glycolide). They observed that increasing the poly(lactide-co-glycolide) content significantly reduced the rapid release of curcumin from the microparticles in buffers with pH 1.2 and 6.8. The optimal formulation, microparticles with a weight ratio of 1:2, demonstrated the sustained release of curcumin, achieving approximately 48% drug release at pH 7.2–7.4 over a 20 h incubation period. In vivo studies showed that orally administered microparticles exhibited superior therapeutic efficacy in alleviating colitis in a UC mouse model compared to free curcumin. These curcumin-loaded microparticles combine the advantages of pH sensitivity, controlled drug release, and effective colon targeting, making them a promising drug carrier for the efficient treatment of UC [60].

Sareen et al. [80] developed pH-sensitive Eudragit^®^-coated chitosan microspheres (Table 1) for delivering curcumin to treat UC. They were prepared using an emulsion crosslinking technique and subsequently coated with Eudragit^®^ S100. The pharmacodynamic performance of the formulation was evaluated in an acetic acid-induced colitis model in mice. X-ray diffraction analysis revealed less intense peaks for the curcumin–chitosan microspheres compared to free curcumin, confirming successful drug encapsulation. Uncoated microspheres displayed a burst release within the first 4 h, whereas the Eudragit^®^ S100-coated microspheres demonstrated controlled release up to 12 h, following the Higuchi release model. In vivo studies showed a significant reduction in the severity and extent of colonic damage with curcumin-loaded microspheres compared to pure curcumin treatment. These findings establish the developed microspheres as a promising system for pH-dependent colon-specific drug delivery in UC therapy [80].

The study of Heikal et al. [81] focused on developing a drug delivery system for effectively treating colitis using a combination of curcumin and mesalamine (Table 1). The approach involved encapsulating the drugs in alginate/chitosan beads coated with Eudragit^®^ S100 to enable colon-targeted delivery. The Eudragit^®^ S100 coating ensured pH-sensitive release, preventing drug release at pH levels below 7, as demonstrated through in vitro release studies simulating the pH conditions of the GIT. The optimized formulation consisted of mesalamine and curcumin as active ingredients, sodium alginate as a gelling agent, chitosan for controlled release, calcium chloride (CaCl_2_) as a crosslinking agent, and Eudragit^®^ S100 as the pH-sensitive coating. Drug release studies revealed minimal release in acidic conditions (6.01 ± 0.04% curcumin and 8.64 ± 0.7% mesalamine at pH 1.2 after 2 h), moderate release at intestinal pH (6.36 ± 0.11% curcumin and 10.45 ± 1.52% mesalamine at pH 6.8 after 4 h), and significant release at colonic pH (85.34 ± 2.3% curcumin and 91.5 ± 1.2% mesalamine at pH 7.4 after 24 h). In vivo studies using an acetic acid-induced colitis model in rats demonstrated that the optimal formulation significantly alleviated colitis symptoms. These findings suggest that the developed hydrogel beads are a promising system for delivering curcumin and mesalamine combinations to effectively treat UC [81].

### 7.15. Mesalamine (Mesalazine)

Cao et al. [82] developed a novel pH/enzyme double-dependent colon-specific mesalamine delivery system (Table 1). It was developed through a two-step process: the preparation of mesalamine-loaded chitosan microparticles and their subsequent coating with a pH-sensitive polymer. The microparticles were synthesized using an emulsion chemical crosslinking technique, and the coating was applied using fluid-bed spray technology. Specifically, 0.1 g of chitosan microparticles were coated with Eudragit^®^ S100 in an aqueous medium, achieving a 20% weight gain. In vitro studies showed minimal mesalamine release in acidic conditions (pH 1.2) and neutral conditions (pH 7.4) over the first 5 h. However, 71% of the drug was released during the following 20 h, demonstrating a controlled release profile. Pharmacokinetic studies in rats revealed a significantly higher area under the plasma concentration–time curve for the coated microparticles, indicating the efficient absorption of mesalamine over a 12 h period. The findings suggest that the mesalamine-coated microparticles can maintain therapeutic drug concentrations in the colon for extended durations, making this system a promising approach for treating colonic diseases [82].

Patil et al. [83] developed mucoadhesive microspheres for colon-targeted drug delivery using a sodium alginate core loaded with mesalamine (Table 1). The core microspheres were coated with Eudragit^®^ S100 via a solvent evaporation technique. The Eudragit^®^ S100 coating delayed drug release until the microspheres reached the colon, where the coating dissolved. The sodium alginate core’s mucoadhesive properties allowed for a prolonged residence time in the colon, enhancing localized drug delivery. The study concluded that combining pH-dependent polymers, Eudragit^®^ S100, and sodium alginate effectively controlled drug release and achieved colon-specific delivery, with the formulation providing the sustained release of mesalamine [83].

Pawar et al. [84] developed a pulsatile controlled-release system using a pH- and time-dependent approach to achieve the colon-specific delivery of mesalamine (Table 1). The system consisted of an enteric-coated capsule with a water-soluble cap and an insoluble body. The drug formulation, comprising mesalamine coated with Eudragit^®^ L100 and S100 copolymers, was loaded into the capsule body and separated from the cap by a hydrogel plug. Upon reaching the small intestine, the enteric coating dissolved, and the hydrogel plug began to swell, accounting for the transit time through the small intestine. Finally, the swollen plug was ejected, enabling the formulation to be delivered to the colon. Once in the colon, the pH-responsive coating further controlled drug release, sustaining it for up to 24 h [84].

### 7.16. Mesalamine and Prednisolone

Saraogi et al. [85] prepared pectin microspheres (Table 1) containing mesalamine and prednisolone using an emulsion dehydration technique and coated them with the pH-sensitive polymer Eudragit^®^ S100 to achieve controlled drug release. The microspheres exhibited an initial burst release, followed by a sustained release of both drugs over 14 h at colonic pH, confirming their pH-dependent release behavior. These findings suggest that Eudragit^®^ S100-coated pectin microspheres of Saraogi et al. could be a promising approach for treating ulcerative colitis [85].

## 8. Marketed pH-Dependent Drug Delivery Systems

pH-dependent drug delivery systems for UC treatment are already in production and successfully available on the market (Table 2). Certain mesalazine formulations are designed with an enteric coating to control the release site of the active pharmaceutical ingredient. For instance, Asacol^®^ (Tillotts Pharma GmbH, Rheinfelden, Germany) and Octasa^®^ (Tillotts Pharma UK Ltd., Wellingore, Lincolnshire, United Kingdom) utilize a methacrylate copolymer coating, Eudragit^®^ S, which dissolves at pH ≥ 7, targeting drug release to the terminal ileum and colon. On the other hand, formulations like Salofalk^®^ (Dr Falk GmBH, Freiburg im Breisgau, Germany), Mesasal^®^ (Aspen Pharmacare, St Leonards, Australia), and Claversal^®^ (Recordati Pharma GmbH, Ulm, Germany) use an Eudragit^®^-L coating that disintegrates at pH ≥ 6, allowing the drug to be released throughout the mid to distal ileum and colon [86]. Another example is Lialda^®^, Nogra Pharma Limited, Dublin, Ireland, a delayed-release tablet containing mesalamine dispersed within a lipophilic matrix, approved in January 2007 for localized action. Additionally, multiparticulate pH-dependent formulations of budesonide, such as Budenofalk^®^ (Dr Falk Pharma GmbH, Freiburg, Germany), Entocort^®^ (Tillotts Pharma AG, Rheinfelden, Germany) and Budesonide MMX^®^ are also commercially available. Budenofalk^®^ incorporates pH-sensitive polymers like Eudragit^®^-RL, Eudragit^®^-S, and Eudragit^®^-L, whereas Entocort^®^ employs delayed-release polymers like ethylcellulose for time-dependent drug release [87]. Budesonide MMX^®^ (Cosmo Pharmaceuticals SpA, Lainate, Italy) represents an innovative once-daily oral formulation of budesonide that employs Multi Matrix (MMX^®^) colonic delivery technology, facilitating the controlled release of budesonide throughout the colon [88]. There is also evidence that novel formulations of budesonide and mesalazine could be cost-effective alternatives compared other oral formulations [89,90,91,92]. Mezavant^®^ (Takeda Pharmaceuticals international AG, Dublin, Ireland) is considered a cost-effective treatment for UC in the UK, Germany, and the Republic of Kazakhstan [90,91,92]. However, in Spain, the lower cost per gram of Salofalk^®^ (Dr Falk Pharma GmbH, Freiburg, Germany) for the treatment of mild–moderate UC, makes this drug more a cost-effective option compared to Mezavant^®^ [93]. In The Netherlands, a study conducted by Gherardi et al. [89] demonstrated that Budesonide MMX^®^ is cost-effective compared to alternative therapies for the second-line treatment of active mild-to-moderate UC.

## 9. Conclusions

The colonic region of the GIT has become a critical focus for drug delivery and absorption, particularly for the topical treatment of UC. Most available treatments for UC aim to alleviate symptoms, with the choice of therapy depending on the disease’s severity. Over recent decades, the development of new colonic drug delivery systems has garnered significant attention as a means to enhance treatment efficacy while minimizing the dosage of active agents, reducing systemic side effects through limited intestinal absorption, and decreasing the frequency of drug administration. This review explores the potential of pH-dependent drug delivery for UC treatment. Various polymers and techniques have been employed to create pH-sensitive drug delivery systems, with researchers striving to encapsulate different drugs. Studies in this field have consistently shown promising outcomes, as demonstrated by the successful commercialization of medical products for colitis treatment. Importantly, research continues to explore new combinations and approaches to identify the most effective systems for delivering multiple drug molecules simultaneously, improving convenience and outcomes for patients. Despite these advances, challenges remain for pharmaceutical scientists. Developing and validating dissolution methods that accurately simulate the colon’s physiological environment while being suitable for the routine industrial evaluation of CDDSs is an ongoing hurdle. Nevertheless, recent progress in the design and development of colon-targeted therapies holds significant promise for introducing safer, more effective treatments into clinical practice.

## Figures and Tables

**Table 1:** List of studies investigating different pH-dependent polymers used in UC treatment.

Author/Ref.	Year	Drug Candidates for Treatment of UC	pH-Dependent Polymer
Vaidya et al. [61]	2009	Metronidazole	Eudragit^®^ S100
Makhlof et al. [62]	2009	Budesonide	Eudragit^®^ S100
Yoo et al. [63]	2015	Budesonide	Eudragit^®^ FS30D
Naeem et al. [64]	2020	Budesonide	Eudragit^®^ FS30D
Turanlı and Acartürk [65]	2022	Budesonide	Eudragit^®^ S100
Alkazzaz et al. [66]	2015	Prednisolone	Eudragit^®^ S100
Liu et al. [67]	2010	Prednisolone	Eudragit^®^ S100
Hashem et al. [68]	2013	Prednisolone	Eudragit^®^ S100
Qu et al. [69]	2021	Budenoside and prednisolone	Eudragit^®^ S100
Shi et al. [70]	2018	Hydrocortisone sodium succinate	poly(ethylene glycol) methyl ether methacrylate
Oshi et al. [71]	2018	Dexamethasone	poly(ethylene glycol) methyl ether methacrylate
Naeem et al. [72]	2014	Coumarin	Eudragit^®^ S100
Tie et al. [73]	2023	Notoginsenoside	Eudragit^®^ S100
Oshi et al. [74]	2021	Cyclosporine	Eudragit^®^ S100
Gao et al. [75]	2021	Iridoid glycosides	Eudragit^®^ S100 and Eudragit^®^ L30D-55
Pande et al. [76]	2020	Flurbiprofen	Eudragit^®^ L 100 and Eudragit^®^ S 100
Huang et al. [77]	2024	Baicalin	Eudragit^®^ S100
Lv et al. [78]	2023	Methotrexate	Eudragit^®^ S100
Gugulothu et al. [79]	2014	Curcumin	Eudragit^®^ S100
Xiao et al. [60]	2015	Curcumin	Eudragit^®^ S100
Sareen et al. [80]	2016	Curcumin	Eudragit^®^ S100
Heikal et al. [81]	2023	Curcumin and mesalamine	Eudragit^®^ S100
Cao et al. [82]	2016	Mesalamine	Eudragit^®^ S100
Patil et al. [83]	2018	Mesalamine	Eudragit^®^ S100
Pawar et al. [84]	2018	Mesalamine	Eudragit^®^ S100 and Eudragit^®^ L100
Saraogi et al. [85]	2023	Mesalamine and prednisolone	Eudragit^®^ S100

**Table 2:** Authorized oral mesalazine and budesonide medicinal products using pH-dependent polymers (examples from Europe and U.S.) [94,95].

INN	Brand Name/Marketing Authorisation Holder	Dosage	Dosage Form	pH-Dependent Polymer
Mesalazine	Colitofalk^®^ (Dr. Falk Pharma GmbH, Freiburg, Germany)	1000 mg 1500 mg	Gastro-resistant granules	Methacrylic acid–methyl methacrylate copolymer (1:1) (Eudragit^®^ L 100)
	Asacol^®^ (Tillotts Pharma GmbH, Rheinfelden, Germany)	800 mg 1600 mg	Modified colon-release tablets	Methacrylic acid–methyl methacrylate copolymer (1:2) (Eudragit^®^ S 100)
	Asacolon^®^ (Tillotts Pharma GmbH, Rheinfelden, Germany)	400 mg 800 mg	Gastro-resistant tablets	Methacrylic acid–methyl methacrylate copolymer (1:2) (Eudragit^®^ S 100)
	Azzavix^®^ (Faes Farma, S.A., Leioa, Spain)	500 mg 1000 mg	Gastro-resistant tablets	Methacrylic acid–methyl methacrylate copolymer (1:1) (Eudragit^®^ L 100) Methacrylic acid–methyl methacrylate copolymer (1:2) (Eudragit^®^ S 100)
	Asamax^®^ Astellas Pharma Sp. z o.o., Warsaw, Poland	400 mg 800 mg	Gastro-resistant tablets	Methacrylic acid–methacrylate–methyl methacrylate copolymer (Eudragit^®^ FS30 D)
	Asamovon^®^ (Tillotts Pharma GmbH, Rheinfelden, Germany)	1600 mg	Modified release tablets	Methacrylic acid–methyl methacrylate copolymer (1:2) (Eudragit^®^ S 100)
	Claversal^®^ (Recordati Pharma GmbH, Ulm, Germany)	500 mg 1000 mg	Modified colon-release tablets	Methacrylic acid–methyl methacrylate copolymer (1:1) (Eudragit^®^ L 100)
	Salofalk^®^ (Dr Falk GmBH, Freiburg im Breisgau, Germany)	250 mg, 500 mg 1000 mg tablets/granules	Delayed-release granules have matrix core. Released in mid ileum to colon	Methacrylic acid–methyl methacrylate copolymer (1:1) (Eudragit^®^ L 100) Methacrylic acid–methyl methacrylate copolymer (1:2) (Eudragit^®^ S 100)
	Octasa^®^ Tillotts Pharma UK Ltd, Wellingore Lincolnshire United Kingdom	400 mg, 800 mg 1600 mg	Modified colon-release tablets	Methacrylic acid–methyl methacrylate copolymer (1:2) (Eudragit^®^ S 100)
	Mezavant^®^ Mezavant XL^®^ (Takeda Pharmaceuticals international AG, Dublin, Ireland)	1200 mg tablets gastro-resistant, prolonged release tablets	MMX Multi Matrix system technology. Released in terminal ileum and entire colon	Methacrylic acid–methyl methacrylate copolymer (1:1) (Eudragit^®^ L 100) Methacrylic acid–methyl methacrylate copolymer (1:2) (Eudragit^®^ S 100)
	Galtasa^®^ (Faes Farma, S.A., Leioa, Spain)	1000 mg	Gastro-resistant tablets	Methacrylic acid–methyl methacrylate copolymer (1:1) (Eudragit^®^ L 100) Methacrylic acid–methyl methacrylate copolymer (1:2) (Eudragit^®^ S 100)
	Delzicol^®^ Allergan Pharmaceuticals International Limited, Madison, NJ, USA	400 mg capsusles	Delayed-release in terminal ileum and colon	Methacrylic acid–methyl methacrylate copolymer (1:2) (Eudragit^®^ S 100)
	Lialda^®^ (Nogra Pharma Limited, Dublin, Ireland)	1200 mg	MMX Multi Matrix system technology. Release in terminal ileum and entire colon	Methacrylic acid–methyl methacrylate copolymer (1:1) (Eudragit^®^ L 100) Methacrylic acid–methyl methacrylate copolymer (1:2) (Eudragit^®^ S 100)
	Mecolvix^®^ (Faes Farma, S.A., Leioa, Spain)	500 mg 1000 mg	Gastro-resistant tablets	Methacrylic acid–methyl methacrylate copolymer (1:1) (Eudragit^®^ L 100) Methacrylic acid–methyl methacrylate copolymer (1:2) (Eudragit^®^ S 100)
	Mecolzine^®^ (Faes Farma, S.A., Leioa, Spain)	500 mg 1000 mg	Gastro-resistant tablets	Methacrylic acid polymer–ethyl acrylate copolymer (1:1) Methacrylic acid–methyl methacrylate copolymer (1:1) (Eudragit^®^ L 100) Methacrylic acid–methyl methacrylate copolymer (1:2) (Eudragit^®^ S 100)
	Mesagran^®^ (Dr. Falk Pharma GmbH, Freiburg, Germany)	500 mg, 1000 mg, 1500 mg, 3000 mg	Gastro-resistant sustained-release granules	Methacrylic acid–methyl methacrylate copolymer (1:1) (Eudragit^®^ L 100)
	Melazine Orion^®^ (Faes Farma, S.A., Leioa, Spain)	500 mg, 1000 mg	Enteric tablet	Methacrylic acid–methyl methacrylate copolymer (1:1) (Eudragit^®^ L 100) Methacrylic acid–methyl methacrylate copolymer (1:2) (Eudragit^®^ S 100)
	Mesavancol^®^ Giuliani S.p.A, Milan, Italy	1200 mg	Modified release tablets	Methacrylic acid–methyl methacrylate copolymer (1:1) (Eudragit^®^ L 100) Methacrylic acid–methyl methacrylate copolymer (1:2) (Eudragit^®^ S 100)
	Osperzo^®^ (Dr. Falk Pharma GmbH, Freiburg, Germany)	1500 mg 3000 mg	Prolonged-release granules	Methacrylic acid–methyl methacrylate copolymer (1:1) (Eudragit^®^ L 100)
	Yaldigo^®^ (Tillotts Pharma GmbH, Rheinfelden, Germany)	1600 mg	Modified release tablets	Methacrylic acid–methyl methacrylate copolymer (1:2) (Eudragit^®^ S 100)
Budesonide	Budenofalk^®^ Budenofalk Uno (Dr Falk Pharma GmbH, Freiburg, Germany)	3 mg capsules 9 mg granules	Garstro-resistant	Methacrylic acid–methyl methacrylate copolymer (1:1) (Eudragit^®^ L 100) Methacrylic acid–methyl methacrylate copolymer (1:2) (Eudragit^®^ S 100)
	Entocort CR^®^ (Tillotts Pharma AG, Rheinfelden, Germany)	3 mg	Garstro-resistant capsules and microgranules	Methacrylic acid–ethyl acrylate copolymer (1:1)
	Cortiment^®^ Cortiment^®^MMX^®^ Ferring Arzneimittel GmbH, Kiel, Germany	9 mg	Prolonged-release tablets	Methacrylic acid–methyl methacrylate copolymer (1:1) (Eudragit^®^ L 100) Methacrylic acid–methyl methacrylate copolymer (1:2) (Eudragit^®^ S 100)

## Abbreviations

The following abbreviations are used in this manuscript:

UC	Ulcerative colitis
IBD	Inflammatory bowel disease
GIT	Gastrointestinal tract
5-ASA	5-aminosalicylic acid
HSS	Hydrocortisone sodium succinate
CsA	Cyclosporine A
BA	Baicalin
MXT	Methotrexate
CDDS	Colon-specific drug delivery system
